# Combination Therapy Is Superior to Sequential Monotherapy for the Initial Treatment of Hypertension: A Double‐Blind Randomized Controlled Trial

**DOI:** 10.1161/JAHA.117.006986

**Published:** 2017-11-18

**Authors:** Thomas M. MacDonald, Bryan Williams, David J. Webb, Steve Morant, Mark Caulfield, J. Kennedy Cruickshank, Ian Ford, Peter Sever, Isla S. Mackenzie, Sandosh Padmanabhan, Gerald P. McCann, Jackie Salsbury, Gordon McInnes, Morris J. Brown, for The British Hypertension Society Programme of Prevention And Treatment of Hypertension With Algorithm‐based Therapy (PATHWAY)

**Affiliations:** ^1^ Medicines Monitoring Unit Division of Molecular and Clinical Medicine School of Medicine Ninewells Hospital & Medical School University of Dundee United Kingdom; ^2^ Institute of Cardiovascular Sciences University College London (UCL) London United Kingdom; ^3^ National Institute for Health Research (NIHR) UCL/UCL Hospitals Biomedical Research Centre London United Kingdom; ^4^ Clinical Pharmacology Unit Centre for Cardiovascular Science Queen's Medical Research Institute University of Edinburgh United Kingdom; ^5^ Research Nurse William Harvey Institute QMUL London United Kingdom; ^6^ Cardiovascular Medicine & Diabetes King's College London United Kingdom; ^7^ Robertson Centre for Biostatistics University of Glasgow United Kingdom; ^8^ Institute of Cardiovascular Medical Sciences University of Glasgow United Kingdom; ^9^ International Institute for Circulatory Health Imperial College London London United Kingdom; ^10^ NIHR Leicester Cardiovascular Biomedical Research Centre Glenfield Hospital Leicester United Kingdom

**Keywords:** angiotensin II receptor blocker, comparative effectiveness, diuretics, renin, treatment effectiveness, Hypertension

## Abstract

**Background:**

Guidelines for hypertension vary in their preference for initial combination therapy or initial monotherapy, stratified by patient profile; therefore, we compared the efficacy and tolerability of these approaches.

**Methods and Results:**

We performed a 1‐year, double‐blind, randomized controlled trial in 605 untreated patients aged 18 to 79 years with systolic blood pressure (BP) ≥150 mm Hg or diastolic BP ≥95 mm Hg. In phase 1 (weeks 0–16), patients were randomly assigned to initial monotherapy (losartan 50–100 mg or hydrochlorothiazide 12.5–25 mg crossing over at 8 weeks), or initial combination (losartan 50–100 mg plus hydrochlorothiazide 12.5–25 mg). In phase 2 (weeks 17–32), all patients received losartan 100 mg and hydrochlorothiazide 12.5 to 25 mg. In phase 3 (weeks 33–52), amlodipine with or without doxazosin could be added to achieve target BP. Hierarchical primary outcomes were the difference from baseline in home systolic BP, averaged over phases 1 and 2 and, if significant, at 32 weeks. Secondary outcomes included adverse events, and difference in home systolic BP responses between tertiles of plasma renin. Home systolic BP after initial monotherapy fell 4.9 mm Hg (range: 3.7–6.0 mm Hg) less over 32 weeks (*P*<0.001) than after initial combination but caught up at 32 weeks (difference 1.2 mm Hg [range: −0.4 to 2.8 mm Hg], *P*=0.13). In phase 1, home systolic BP response to each monotherapy differed substantially between renin tertiles, whereas response to combination therapy was uniform and at least 5 mm Hg more than to monotherapy. There were no differences in withdrawals due to adverse events.

**Conclusions:**

Initial combination therapy can be recommended for patients with BP >150/95 mm Hg.

**Clinical Trial Registration:**

URL: http://www.ClinicalTrials.gov. Unique identifier: NCT00994617.

**Clinical Perspective**

**What Is New?**

- Initial combination therapy for hypertension achieved target blood pressure in twice as many participants as initial monotherapy, without any difference in withdrawals due to adverse events.
- The blood pressure reduction by losartan and hydrochlorothiazide was greatest in the top and bottom tertiles, respectively, of plasma renin.
- The more effective monotherapy, identified by renin profiling or sequential comparison of the 2 drugs, reduced systolic blood pressure, on average, by 5 mm Hg less than initial combination therapy.

**What Are the Clinical Implications?**

- Initial combination therapy can be recommended for the treatment of blood pressure levels >150/95 mm Hg.
- Initial combination therapy obviates the routine measurement of plasma renin, but extreme values can help the selection of monotherapy when desired.

Most hypertensive patients do not have their blood pressure (BP) controlled by monotherapy. Yet combinations of drugs are not commonly used as initial therapy for hypertension despite studies that suggest the advantages of this approach.^1^, ^2^, ^3^ Initial treatment of hypertension with combination therapy has been discouraged because of concern about excessive reduction in BP, increased side effects, and the difficulty of attributing adverse events to 1 drug. However, the alternatives also have risks and downsides. The most common practice, long recommended by guidelines, is to start with 1 drug and add another. But this could result in patients continuing to take a drug that is ineffective. Some guidelines recommend tailoring initial therapy to patient characteristics such as age and ethnicity, which may be surrogates for plasma renin status.^4^, ^5^ An alternative is sequential monotherapy to find each patient's best monotherapy, but this takes time and endurance, during which uncontrolled hypertension is not without risk.^6^

Evidence that the prompt versus delayed control of BP was of benefit came initially from the VALUE (Valsartan Antihypertensive Long‐term Use Evaluation) trial. In that study, a BP difference of −3.8 mm Hg in the first 3 months of the trial was associated with fewer cardiovascular events, particularly stroke and mortality.^7^ Recent observational analyses have confirmed that delays in achieving BP control are not benign and that rapid BP control is important.^8^, ^9^

These observations have led to recommendations in some guidelines to consider initial combination therapy in patients with BP >160/100 mm Hg, but this practice has not been widely adopted. We thought practitioners might be persuaded by a finding that superior BP control by initial combination was sustained—indeed, this possibility was suggested by the results of the VALUE^7^ and ASCOT (Anglo Scandinavian Cardiac Outcomes Trial)^10^ studies. In these studies, the BP lowering in participants randomized to the less effective of 2 treatments early in the study “never caught up” with that achieved by the other group, despite the former eventually receiving more drug therapy. Furthermore, our previous study of dual therapy versus monotherapy suggested that participants who began initial dual therapy always appeared to have better BP control than the monotherapy group, perhaps because combination therapy attenuated compensatory changes induced by monotherapy.^11^

The objective of PATHWAY‐1 (Prevention And Treatment of Hypertension With Algorithm‐based Therapy ‐ study 1) was to test the superiority of home systolic BP (HSBP) control of initial combination therapy compared with the more conventional practice of initial monotherapy. Half of the patients received combination from the start, and all participants received combination therapy after the first 4 months; this design allowed us to rigorously test the “never‐catch‐up” hypothesis. If this were true, we would find a difference in BP at 8 months despite both groups receiving the same therapy during the previous 4 months. We powered the study to detect a 4‐mm Hg difference in HSBP, as we deemed this difference would be clinically important. An important secondary objective of this design was to test the predictors (age, renin^12^) of response to treatment, which predicts the response to monotherapy but not, we hypothesized, to combination therapy. Although we evidently expected the average BP response to combination therapy to be superior to the average response to monotherapy, the crossover design of phase 1 enabled us to address the question of whether personalized monotherapy achieves a BP response that is not only superior to average but also comparable to the effect of combination.

## Methods

### Setting

The British Hypertension Society Research Network of academic investigators recruited participants from both primary and secondary care.

### Study Design

Patients who were aged 18 to 79 years with a diagnosis of essential hypertension, who had systolic BP >150 mm Hg or diastolic BP >95 mm Hg, and who were either never treated or who had received 1 drug class in the previous year were eligible for inclusion in PATHWAY‐1. This was a parallel‐group, randomized, double‐blind, phase 4 trial conducted in 11 secondary and 2 primary care centers in the United Kingdom. A full list of inclusion and exclusion criteria is provided in Table S1.

**Figure 1:**
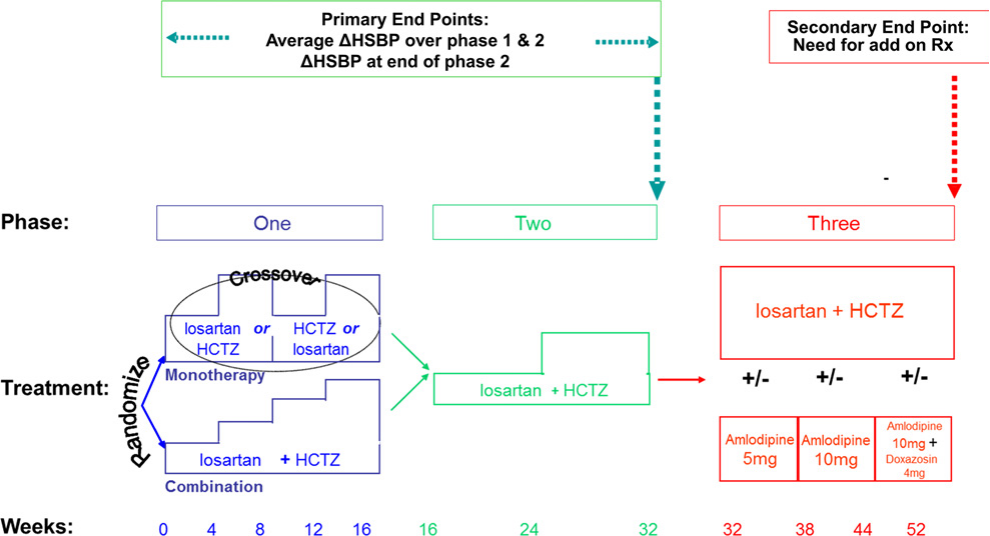
Schematic of the study showing drug administration in each of the 3 phases. HCTZ indicates hydrochlorothiazide; HSBP, home systolic blood pressure.

Participants were enrolled between February 2010 to November 2013; the study ended (last patient completed the study) in December 2014. The study design and rationale have been published.^13^ A study schematic is shown in Figure 1 (a more detailed diagram is shown in Figure S1).

After a 4‐week, single‐blind, placebo run‐in during which any previous antihypertensive monotherapy was withdrawn, there were 3 sequential phases of active treatment during which investigators and participants were blinded to the initial random assignment. In the first phase, weeks 0–16, half of the participants started monotherapy with either losartan or HCTZ (hydrochlorothiazide), and half started a combination of losartan plus HCTZ. In the second phase (weeks 16–32), all participants received the same combination of losartan plus HCTZ. In the third phase, patients received additional open‐label medication with amlodipine and/or doxazosin, dependent on whether their BP was controlled and whether they tolerated the amlodipine. The primary end points were measured at the end of the second phase; the third phase allowed extended assessment of the sustained effect of the difference in randomly assigned drugs in the first phase without withholding clinically desirable treatment escalation.

Eligible participants were randomly assigned at a ratio of 1:1 between monotherapy and combination therapy, but in practice, randomization was 1:1:2 to treatment with 50 mg losartan, or 12.5 mg HCTZ, as first monotherapy, versus 50 mg losartan plus 12.5 mg HCTZ combination therapy. In the monotherapy arms, there was forced dose‐doubling after 4 weeks of treatment, then participants crossed over at 8 weeks to the alternative drug at the lower dose, which was again force‐titrated to twice the dose after a further 4 weeks. In the combination arm, the successive dose permutations at every 4 weeks after initial assignment were losartan/HCTZ 50/25, 100/12.5, and 100/25 mg. After 16 weeks (start of phase 2), all participants received the combination of losartan 100 mg plus HCTZ 12.5 mg. At week 24, all participants were force‐titrated to losartan 100 mg plus HCTZ 25 mg. At week 32 (start of phase 3), amlodipine was added if systolic BP was >140 mm Hg or diastolic BP was >90 mm Hg. There were 2 subsequent opportunities for addition or titration of amlodipine, to 10 mg, with doxazosin 4 to 8 mg as a permitted alternative or addition, as required. The study ended at 52 weeks.

Losartan and HCTZ tablets were reencapsulated in identical gelatin capsules backfilled with microcrystalline cellulose and magnesium stearate, which was also used for the matching placebo capsules. All patients received 2 capsules throughout phase 1, in double‐dummy fashion, to maintain masking in the combination and monotherapy groups. Emergency code breaks via the central Interactive Voice Response System were available if deemed absolutely necessary. In phases 2 and 3, losartan and HCTZ were provided in a single tablet, the identity of which was known to investigators and patients; however, masking of the initial assignment was maintained until database lock.

Adherence to study medication was assessed by return tablet count.

Home BP was measured using the Microlife WatchBP Home monitor on the last 4 days before each clinic visit. This was used instead of ambulatory BP monitoring to improve patient acceptability and to minimize dropouts.

Readings were performed in triplicate, morning and evening, after 10 minutes of seated rest. Clinic BP measurements were recorded in triplicate after 10 minutes of seated rest taken at each study visit using the participant's own Microlife monitor. Plasma renin mass was measured at baseline, using the Diasorin Liaison automated chemiluminescent immunoassay for direct renin.^14^ A full biochemical series was taken at regular intervals for safety.

A cardiac magnetic resonance imaging substudy was undertaken at 3 of the participating centers where magnetic resonance imaging capacity for research studies existed (Cambridge, Leicester, and Dundee) to measure left ventricular (LV) mass at baseline and after treatment for 12 months. In these centers, all participants were offered the magnetic resonance imaging substudy until at least 60 participants had been recruited. All cardiac magnetic resonance imaging data were collected using a common protocol^15^ and processed by clinicians (S.N., G.P.M.) blinded to treatment and other demographics at a central site at Leicester using manual contours drawn with CMR42 software (Circle Cardiovascular Imaging) with exclusion of the papillary muscles and trabeculations and normalized for body mass.^16^

All adverse events considered related to treatment were recorded on the electronic case record form and coded by the data management center on the basis of the medical dictionary for regulatory activities. Serious adverse events were documented and reported to the chief investigator and to regulatory authorities, in accordance with local and national requirements.

The original protocol prespecified the time of the primary end point at the end of phase 2, namely, 32 weeks after randomization, at which time all patients were receiving the same therapy. The statistical analysis plan, published before data lock and unblinding, described 2 hierarchical co–primary end points.^13^ The first was the mean reduction from baseline of HSBP over phases 1 and 2, testing for superiority between the losartan plus HCTZ (initial combination) group and the mean of each monotherapy arm. The second primary end point (tested only if the first hypothesis was positive) was the reduction from baseline in HSBP at week 32, a point in the study when all participants were receiving the same treatment.

Secondary end points included the reductions in HSBP at 52 weeks and the reductions in clinic systolic BP at the same time points as home BP recordings. To test the “AB/CD” rule—recommending that an angiotensin‐converting enzyme inhibitor or an angiotensin receptor blocker (ARB) or beta‐blocking drug be prescribed initially to younger patients and a calcium antagonist or diuretic be prescribed to older patients^4^, ^17^—and whether this rule could be improved by measurement of plasma renin,^18^ we examined the effect of baseline variables, particularly age and plasma renin, on response to each monotherapy at the end of phase 1. We also investigated the proportion of participants who achieved target HSBP (<135 mm Hg) and the response rates, defined as the proportion of participants with systolic BP <135 mm Hg or reduction >10 mm Hg. The proportions of participants with adverse events and withdrawals were compared between treatment groups.

In the crossover substudy, we identified the monotherapy that provided the best response in phase 1 for each participant. We prespecified analyses to examine predictors of the better treatment, including age >55 or <55 years (this is the cut point for choosing between A and D in some guidelines^17^) and tertiles of plasma renin.

The study was approved by Cambridgeshire Research Ethics Committee (09/H0308/132), and all participants gave written informed consent.

### Statistical Analyses

A sample size of 268 in each group had 90% power to detect a difference in HSBP means of 4 mm Hg, assuming a common SD of 12 mm Hg using a 2‐group *t* test with α=0.01. We hypothesized 4 mm Hg because this was the difference in systolic BP between arms in the VALUE study at 3 months that was associated with significant excess mortality.^7^

All analyses followed a prespecified statistical analysis plan.

We used the arithmetic mean of last 18 home BP measurements before a study visit to calculate home BP. If >18 measurements were obtained, the earliest recorded readings were discarded. For most patients, we used all readings on days 1 to 3 before the visit, but if any of these were missing, readings from day 4 before the visit were added. The minimum number of measurements required for a valid assessment of home BP was 6, and if there were <6 readings, we declared the observation missing.

We used mixed‐effect models with unstructured covariances for repeated measures within a patient. These models provide valid tests of hypotheses when observations are missing at random. We adjusted for prespecified baseline covariates (sex, age, height, weight, smoking history, whether or not the participant had been treated for hypertension before the baseline visit, and the baseline value of the outcome being analyzed). Least squares means estimated from these models are presented. BP control and response rates were analyzed using logistic regression models, which also included the baseline covariates. Comparisons of adverse event rates between treatments were conducted using χ^2^ tests, and Fisher exact *P* values are given.

Intention‐to‐treat analyses excluded only those participants with no primary outcome data at any follow‐up visit. Other participants with missing data were included, and we assumed that data were missing at random (ie, its absence was unrelated to the unobserved value).

The investigators and all authors had sole discretion in data analysis and interpretation, writing of the report, and the decision to submit for publication.

## Results

**Figure 2:**
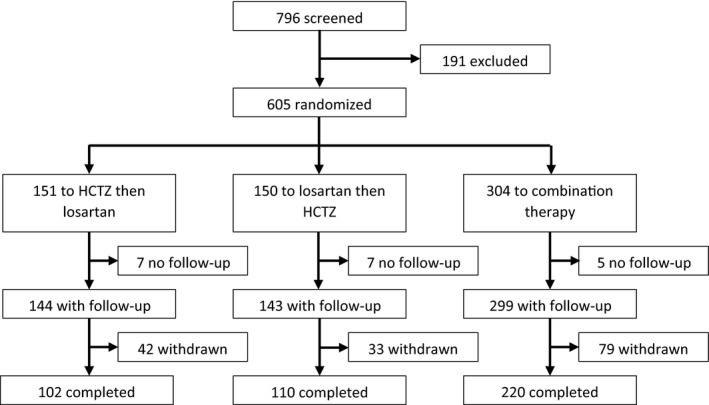
CONSORT (Consolidated Standards of Reporting Trials) diagram showing the patients screened, randomized, and analyzed. Note that withdrawn participants include some patients who completed the protocol but had major protocol deviations or who had incomplete data for the primary outcome. “Withdrawn” equals not eligible for per protocol analysis. The breakdown of withdrawals is shown in Table S3. HCTZ indicates hydrochlorothiazide.

We screened 796 patients and randomized 605 participants, 301 to initial monotherapy and 304 to initial combination therapy; the intention‐to‐treat analysis included 287 and 299 participants, respectively, in these 2 groups (Figure 2).

Table 1 shows the baseline characteristics of participants in the study and indicates that the participants receiving combination therapy were similar to those in each monotherapy arm.

**Table 1:** Patient Demographics at Baseline

	Combination Therapy	Monotherapy (HCTZ First)	Monotherapy (Losartan First)
Age (y), mean (SD)	54.3 (12.1)	53.6 (11.9)	54.5 (12.2)
Female, n (%)	109 (36.5)	51 (35.4)	45 (31.5)
Weight, mean (SD)	86.9 (17.6)	87.5 (17.2)	86.8 (16.5)
BMI, mean (SD)	29.5 (5.6)	29.8 (5.7)	29.0 (5.0)
Diabetic, n (%)	23 (7.7)	9 (6.3)	14 (9.8)
Current smoker, n (%)	23 (7.7)	18 (12.5)	15 (10.5)
Former smoker, n (%)	102 (34.1)	53 (36.8)	47 (32.9)
Never smoked, n (%)	174 (58.2)	73 (50.7)	81 (56.6)
Never previously treated for hypertension, n (%)	135 (45.2)	64 (44.4)	70 (49.0)
Blood pressure, mm Hg			
Home systolic, mean (SD)	152.1 (11.2)	151.8 (13.4)	152.2 (12.1)
Diastolic, mean (SD)	93.2 (9.9)	93.4 (103)	93.6 (8.3)
Clinic systolic, mean (SD)	158.5 (12.0)	158.0 (13.2)	156.3 (12.5)
Diastolic, mean (SD)	98.3 (10.0)	99.3 (10.5)	98.6 (10.0)
Renin, median (IQR)	12 (6–19)	13 (6–22)	12 (7–23)

BMI indicates body mass index; HCTZ, hydrochlorothiazide; IQR, interquartile range.

**Figure 3:**
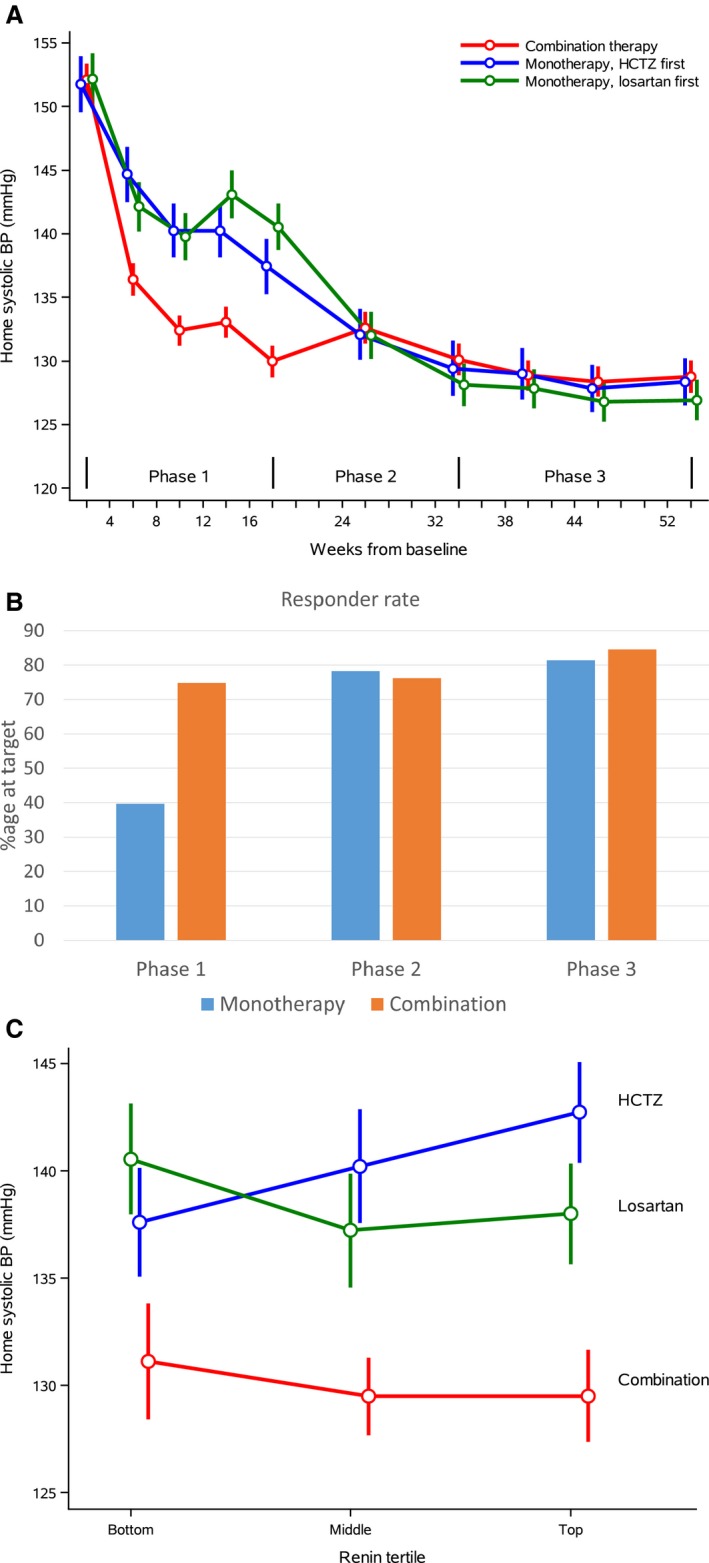
Home systolic BP. A, Means at each visit in each of the monotherapy and combination therapy arms. Unadjusted mean home systolic BP (95% confidence interval) at each visit. B, Responder rates at the end of each phase. C, Means measured on maximum dose of combination therapy and each monotherapy by tertiles of renin mass measured before treatment. Unadjusted mean home systolic BP by renin tertile. BP indicates blood pressure; HCTZ, hydrochlorothiazide.

The mean adjusted change in HSBP from baseline over weeks 0 to 32 was −19.7 mm Hg (95% confidence interval [CI], −20.7 to −18.7) in the initial combination group, and −14.8 mm Hg (95% CI, −15.8 to −13.8) across the monotherapy group (Figure 3A and Table 2). The difference between groups (the primary end point) was −4.9 mm Hg (95% CI, −6.0 to −3.7) and was significant at *P*<0.001 in favor of combination therapy, permitting testing of the second hierarchical primary end point prespecified in the protocol. At the end of phase 2, the mean adjusted reduction in HSBP from baseline was −22.2 mm Hg (95% CI, −23.6 to −20.8) in the initial combination group and −23.4 mm Hg (95% CI, −24.8 to −22.0) in participants receiving initial monotherapy. The difference between groups for this hierarchical primary end point was +1.2 mm Hg (95% CI, +2.8 to −0.4; *P*=0.1). Clinic systolic BP largely mirrored the home BP readings (Table 2); of note was the >10‐mm Hg lower clinic BP in those on combination therapy averaged over phase 1. Table S2 shows the home and clinic BP data split by study week, and Table S3 shows data split by morning and evening home BP readings. The BP‐lowering effects of medication were similar or lower in the evening, suggesting that the trial medication lowered BP throughout the day.

**Table 2:** HSBP Results (Intention‐to‐Treat and Per‐Protocol Analyses) for Primary End Point (Average of Weeks 4–32) and Hierarchical Co–Primary End Point at Week 32

	Blood Pressure (95% CI), mm Hg		Change From Baseline		Difference	*P* Value
	Combination Therapy (n=299)	Monotherapy (n=287)	Combination Therapy	Monotherapy		
HSBP						
Average over phases 1 and 2	132.4 (131.4–133.4)	137.3 (136.3–138.2)	−19.7 (−20.7 to −18.7)	−14.8 (−15.8 to −13.8)	−4.88 (−6.04 to −3.73)	<0.001*
End of phase 2	129.8 (128.4–131.3)	128.6 (127.2–130.0)	−22.2 (−23.6 to −20.8)	−23.4 (−24.8 to −22.0)	1.22 (−0.38 to 2.82)	0.1†
Average over phase 1	133.0 (132.0–134.0)	141.0 (140.0–142.0)	−19.0 (−20.0 to −18.0)	−11.1 (−12.0 to −10.1)	−7.97 (−9.14 to −6.81)	<0.001
Best response in phase 1	127.0 (125.8–128.1)	133.7 (132.5–134.8)	−25.1 (−26.2 to 23.9)	−18.4 (−19.5 to −17.3)	−6.70 (−8.04 to −5.37)	<0.001
Average over study (phases 1–3)	131.1 (130.2–132.0)	134.0 (133.1–134.9)	−20.9 (−21.9 to −20.0)	−18.0 (−18.9 to −17.1)	−2.90 (−3.99 to −1.82)	<0.001
Home diastolic BP						
Average over phases 1 and 2	82.6 (81.9–83.2)	85.8 (85.2–86.5)	−10.8 (−11.4 to −10.1)	−7.5 (−8.1 to −6.9)	−3.27 (−4.01 to −2.54)	<0.001
End of phase 2	81.1 (80.2–82.0)	80.6 (79.6–81.5)	−12.2 (−13.2 to −11.3)	−12.8 (−13.7 to −11.9)	0.55 (−0.49 to 1.59)	0.301
Average over phase 1	82.9 (82.3–83.6)	88.1 (87.4–88.7)	−10.4 (−11.0 to −9.8)	−5.3 (−5.9 to −4.6)	−5.14 (−5.88 to −4.40)	<0.001
Average over study (phases 1–3)	81.9 (81.3–82.5)	83.9 (83.3–84.5)	−11.5 (−12.1 to −10.9)	−9.5 (−10.1 to −8.9)	−2.00 (−2.71 to −1.30)	<0.001
Clinic systolic BP						
Average over phases 1 and 2	133.1 (131.9–134.3)	139.9 (138.7–141.1)	−24.8 (−26.0 to −23.5)	−18.0 (−19.2 to −16.8)	−6.79 (−8.21 to −5.38)	<0.001
End of phase 2	129.2 (127.4–131.1)	130.0 (128.1–131.8)	−28.6 (−30.4 to −26.7)	−27.9 (−29.7 to −26.1)	−0.71 (−2.79 to 1.37)	0.504
Average over phase 1	134.0 (132.8–135.3)	144.1 (142.9–145.3)	−23.8 (−25.1 to −22.6)	−13.7 (−15.0 to −12.5)	−10.1 (−11.5 to −8.63)	<0.001
Average over study (phases 1−3)	131.6 (130.5–132.7)	136.0 (134.9–137.1)	−26.3 (−27.4 to −25.1)	−21.8 (−23.0 to −20.7)	−4.41 (−5.71 to −3.10)	<0.001
Clinic diastolic BP						
Average over phases 1 and 2	84.7 (83.9–85.5)	88.9 (88.2–89.7)	−13.9 (−14.7 to −13.1)	−9.7 (−10.5 to −9.0)	−4.20 (−5.09 to −3.30)	<0.001
End of phase 2	82.6 (81.4–83.8)	82.9 (81.7–84.0)	−16.0 (−17.2 to −14.9)	−15.8 (−16.9 to −14.6)	−0.27 (−1.60 to 1.05)	0.686
Average over phase 1	85.2 (84.4–86.0)	91.5 (90.7–92.3)	−13.4 (−14.2 to −12.6)	−7.1 (−7.9 to −6.3)	−6.31 (−7.23 to −5.39)	<0.001
Average over study (phases 1–3)	83.9 (83.1–84.6)	86.5 (85.8–87.2)	−14.8 (−15.5 to −14.0)	−12.1 (−12.9 to −11.4)	−2.64 (−3.49 to −1.78)	<0.001

Least squares means from mixed effects models adjusted for baseline covariates. BP indicates blood pressure; HSBP, home systolic blood pressure.

*^,†^Hierarchical primary end points, the second tested only if the first is significant.

By the end of phase 3, ≥75% of participants in both groups were at target (Table S4), with no difference between groups at the end of either phase 2 or 3. In contrast, only 40% of responses to initial monotherapy were controlled at target at the end of phase 1 (Figure 3B), increasing to 50% of all responses to monotherapy (ie, on either losartan or HCTZ; Tables S5 and S6). Post hoc analysis identified only 35 participants whose initial monotherapy was their best treatment (Table S7).

In the monotherapy arm crossover, losartan and HCTZ were the more effective drugs in younger and older participants, respectively, but the age effect was modest, and losartan 100 mg was more effective than HCTZ 25 mg in most participants aged >55 years (Table 3). Participants in the top renin tertile responded by 3.7 mm Hg more to losartan and by 4.3 mm Hg less to HCTZ than those in the lowest tertile, an overall difference between treatments of 8 mm Hg (Table 3, *P*<0.001). In contrast, the response to combination therapy was independent of renin tertile and, on average, 5 mm Hg greater than the response to the best drug for each tertile (Figure 3C). There was a greater response to all drugs, especially losartan, in women. Previous treatment favored response to combination, whereas monotherapy was more effective in participants who were naïve to treatment.

**Table 3:** Predictors of BP Response on Each Treatment in Phase 1

	Randomized Initial Treatment					
	Combination (n=299)		HCTZ (n=144)		Losartan (n=143)	
	Difference (95% CI)	*P* Value	Difference (95% CI)	*P* Value	Difference (95% CI)	*P* Value
Top vs bottom renin tertile*	−1.41 (−3.52 to 0.71)	0.2	4.31 (2.26–6.35)	<0.001	−3.71 (−5.70 to −1.71)	<0.001
Aged >55 vs ≤55 y*	1.45 (−0.29 to 3.19)	0.1	−2.94 (−4.73 to −1.15)	0.001	−1.89 (−3.62 to −0.16)	0.03
Renin (per 10‐fold increase)	−1.80 (−4.75 to 1.16)	0.24	4.96 (2.12–7.80)	<0.001	−3.70 (−6.43 to −0.97)	0.008
Age (per 10 y)	0.13 (−0.85 to 1.12)	0.8	−0.97 (−1.98 to 0.04)	0.06	−0.20 (−1.18 to 0.77)	0.7
Baseline HSBP	0.29 (0.22–0.36)	<0.001	0.48 (0.42–0.54)	<0.001	0.55 (0.48–0.61)	<0.001
Never vs previously treated	1.83 (−0.41 to 4.08)	0.1	−3.01 (−5.26 to −0.77)	0.009	−2.85 (−4.96 to −0.73)	0.009

	Effects Not Significantly Different Between Treatment Arms	
Male vs female	2.40 (0.54–4.26)	0.01
Weight (per 10 kg)	0.17 (−0.27 to 0.60)	0.5
Current smoker vs never	1.20 (−1.08 to 3.48)	0.3
Former smoker vs never	2.00 (0.50–3.51)	0.009

Estimates of baseline covariate effects from multivariate models. BP indicates blood pressure; CI, confidence interval; HCTZ, hydrochlorothiazide; HSBP, home systolic blood pressure.

*Prespecified categorization of age and renin, which were used as continuous variables for covariate adjustments in all other analyses.

LV mass was measured in 85 patients (41 on combination therapy and 44 on monotherapy [22 in each monotherapy arm]). LV mass normalized for body surface area was significantly reduced between baseline and the end of the study (−10.4%, *P*<0.001) but was not different between the combination and sequential monotherapy arms of the study.

### Adverse Effects

Adverse and serious effects were similar with combination therapy or monotherapy overall (Table S8). There were more reports of symptoms suggesting hypotension on combination therapy in phase 1 (combination 25.0% versus monotherapy 13.6%, *P*<0.001) and on monotherapy in phase 2 (combination 11.4% versus monotherapy 18.0%, *P*=0.04), but these did not result in significantly increased withdrawals due to adverse effects. Table S9 shows adverse events that occurred in at least 5% of participants on any treatment or that were significantly different between treatments. Some participants were withdrawn or discontinued or did not have sufficient data for full analysis (Table S10). Figure S2 shows a Kaplan–Meier survival analysis of time to withdrawal due to adverse event. It also shows survival to add‐on therapy in phase 3 of the study.

## Discussion

We found a large average difference in HSBP in favor of initial use of combination therapy compared with initial monotherapy across the first 8 months of the study (phases 1 and 2); there was no discernible subset of patients in whom this difference was not observed. It was largely due to the superiority of combination therapy in the first 16 weeks (phase 1), by margins of almost 8 and 10 mm Hg, respectively, for home and clinic systolic BP. Although combination therapy is expected to show superior efficacy and is recommended for stage 2 hypertension by some guidelines, this has not yet become the norm in routine practice. Consequently, we incorporated a number of planned measurements in the study design that we believed, if positive, would help to change practice.

The first of the additional measurements was the co–primary end point of HSBP at 32 weeks, the prespecified time point for evaluation of the never‐catch‐up hypothesis that initial less intense BP control results in subsequently poorer BP control. This hypothesis was rejected. Never catching up in the ASCOT and VALUE trials, from which the hypothesis arose, may be more a consequence of suboptimal treatments being combined rather than optimal treatments being started sequentially.^7^, ^10^ In contrast, our other original comparisons provide strong support for the rationale of initial combination. A unique feature of PATHWAY‐1 is the “trial within a trial” of a randomized monotherapy crossover trial, the aims of which were to ensure that each participant's best therapy at maximal force‐titrated dose was compared with combination therapy. Several clear‐cut, connected findings emerged. First, we established that among the baseline predictors of BP response to each drug, plasma renin provided a measure with substantial and significant differences in response to losartan and HCTZ between the outer tertiles (BP lowering by HCTZ was greatest in the low‐renin tertile and by losartan in the high‐renin tertile). Second, and in marked contrast to the monotherapy responses, with combination therapy, BP reduction did not vary among tertiles of renin or with other predictors. The important practical inference is that initial combination is not only effective but uniformly effective, reducing the heterogeneity in response—as illustrated by the 97% responder and 75% control rates (Table S3), with odds ratios of 8 and 5 compared with monotherapy. Third, as graphically illustrated in Figure 3C, initial combination therapy is not only uniformly effective and superior to monotherapy but is uniformly superior to personalized monotherapy, whether achieved by prediction of each patient's best drug or by systematic crossover between the options. Even if, within the environment of a trial—with its forced dose‐drug titrations and fixed visit appointments—BP in the monotherapy arm caught up with initial combination by 32 weeks, it is reasonable to suggest the marked advantage for everyday practice of a regimen that achieves target BP in the shortest possible time and the least number of visits.

These findings make a strong case for considering initial combination therapy to be more effective than current practice. Moreover, they are supported by prospective, double‐blind data to combat the other main factor blocking uptake of initial combination: concern about adverse events. There was no difference in the overall rate between groups. The slight excess in phase 1 of symptoms associated with better BP reduction did not lead to an excess of withdrawals from the initial combination group.

These aspects demarcate PATHWAY‐1 from the shorter term, largely commercial studies comparing components of fixed‐dose combinations, undertaken for registration of the combinations. Most of these studies used lower relative doses of one or both components than we did, and none provided participants with the opportunity to receive both components at maximal dose and to be compared both with matched patients starting on combination and with their own subsequent response to the combination. Two larger and longer term studies are sometimes cited to support the value of initial combination: the prospective cluster‐randomized STITCH (Simplified Treatment Intervention to Control Hypertension) study and retrospective Wald meta‐analysis.^19^, ^20^ Neither, however, has led to a change in practice, probably because of the limitations imposed by cluster randomization and retrospective analysis and because neither could address the question of adverse events. The STITCH study authors considered that superior BP reduction by initial combination in their study was attributable to their use of fixed‐dose combinations (largely different from the individual drugs used in their initial monotherapy arm) and, consequently, better adherence. Our study was not intended to address this issue but incidentally appears to refute it because average HSBP of the 300 patients in the initial combination arm was virtually identical at 16 and 32 weeks (130.0 and 130.2 mm Hg, respectively) when they were receiving losartan 100 mg and HCTZ 25 mg as separate or single tablets.

The number of participants required to answer the primary hypothesis for PATHWAY‐1 and the median age of 55 years at recruitment gave us the opportunity to prospectively test the age dependence of response underpinning some guidelines, such as National Institute for Health and Care Excellence/British Hypertension Society and the AB/CD rule.^17^ These guidelines predict that drugs blocking the renin–angiotensin system are more effective for younger patients, whereas the reverse is true in older patients, who tend to have lower renin. The continuous correlation between baseline renin and BP response, also seen in our other 2 PATHWAY studies,^21^, ^22^ suggests that there may be outer extremes at which renin predicts extreme responses; indeed, this was the case for our patients responding as well to initial HCTZ or losartan as to their combination (Table S7). But overall, we can conclude that combination trumps stratification, that initial combination trumps initialled (=personalised) monotherapy. Indeed, our crossover study has weakened the case for considering that 1 monotherapy can be selected with confidence that it will be effective. The renin profiling served rather to underpin previous extensive literature that divides the major antihypertensive drugs into 2 complementary categories, targeting either the renin‐angiotensin system or sodium balance; dual action is required in most patients precisely to prevent the one pressor system compensating for blockade of the other.^17^, ^23^, ^24^, ^25^

A limitation of our study is that although larger and longer than most studies comparing combination to monotherapy, results are limited to 1 year and cannot address the impact on morbidity and mortality. In the VALUE study, a 3.8‐mm Hg lower clinic systolic BP in the first 3 months of the study translated into a significant increase in the primary end point driven mainly by mortality and stroke and was borderline for increased myocardial infarction. In our study, clinic systolic BP was 10.1 mm Hg lower in the first 2 months and 6.8 mm Hg on average lower over the first 4 months on combination therapy versus sequential monotherapy, and it is likely that such differences applied to a large population would be clinically important, especially those with similar cardiovascular risk, as participate in morbidity–mortality studies. The BP Lowering Treatment Trialists’ Collaboration found that the relative risk reduction of BP lowering was similar for low‐ and higher risk individuals.^26^ It is likely that significant proportional reductions in cardiovascular events would accrue from treating all hypertensive patients with initial combination therapy. Some reviewers argued that more powerful ARBs have been identified since our study was initiated, citing a meta‐analysis of azilsartan,^27^ or that HCTZ 25 mg is a paltry and unacceptable diuretic because in combination with this same ARB, it reduced systolic BP by 5.6 mm Hg less than a combination with chlortalidone, weighting a meta‐analysis of thiazide‐like versus thiazide‐type diuretics.^28^, ^29^ There is a paucity of outcome data for the latter at their widely used modern doses. These are potentiated by ARBs, with which they are frequently combined to block the compensatory effect of renin–angiotensin stimulation.^19^, ^22^ In the only randomized morbidity–mortality study to demonstrate superiority of an ARB to an active control, losartan was combined with HCTZ 12.5 to 25 mg in most participants.^30^ In a retrospective analysis of a nonrandomized, unmatched comparison of HCTZ and chlortalidone, the event rate was lower in patients receiving chlortalidone (>50 mg in 48%, specialist intervention in 84%) rather than HCTZ (>50 mg in 28%, specialist intervention in 45%).^31^ Not all international guidelines have been convinced of the superiority of thiazide‐like over thiazide‐type diuretics, and these have been compared directly for >12 weeks in <100 patients.^29^, ^32^

A strength of the PATHWAY studies is the use of home BP monitoring as the primary outcome measure. Measured over a number of days and averaged, this value is a better prognostic marker of cardiovascular disease than clinic pressure and avoids misclassification due to the “white coat effect” seen with clinic measures.^33^ Although some guidelines now recommend 24 ambulatory blood pressure measurements for initial diagnosis, this is not a practical alternative to HBPM for routine monitoring of treatment, and within a trial environment, the use of serial ambulatory blood pressure monitoring for primary outcome measurements can compromise power by increasing dropout rates. In addition to use of HBPM, we conducted a substudy assessing LV mass. Consistent with the large reductions in BP over phases 1 and 2 in the combination therapy group and over phase 2 in the monotherapy group, our substudy found >10% reduction in LV mass, which compares favorably with the reduction seen in other studies.^18^ The absence of difference in LV mass between groups confirms rejection of the never‐catch‐up hypothesis and the rapidity with which cardiovascular risk (for which LV mass is the best surrogate) can be reversed by good BP control.

The British Hypertension Society Research Network took a lead role in the design of ACCELERATE (Aliskiren and the Calcium Channel blocker Amlodipine combination as an initial treatment strategy for hypertension control),^9^ a previous trial addressing questions similar to those in PATHWAY‐1. That study was unable to address the question of individual variability and recruited a high proportion of patients on multiple previous drugs. We aimed this time at 50% of naïve patients and achieved 45%, which is rare in studies of this size; the remaining patients had received just 1 previous drug class. Taken together, the present study and the ACCELERATE study^9^ provide a reasonable basis to suggest that the benefits of combination therapy over monotherapy are generalizable. The present study used losartan and HCTZ, whereas ACCELERATE used aliskiren and amlodipine, and both studies demonstrated the superiority of initial treatment with combination therapy. Many combination tablets cost no more than the single components. Whether further reduction of BP by combination therapy is cost‐effective and advantageous in the 40% of patients at target on monotherapy is a matter of current interest on which forthcoming guidelines are likely to have a view.^34^ Initial combination may be favored by those who consider undertreatment to be a more prevalent problem than overtreatment.

## Sources of Funding

The study was funded by a special project grant from the British Heart Foundation (number SP/08/002). Further funding was provided by National Institute of Health Research Comprehensive Local Research Networks. Williams, Sever, Caulfield and Brown are National Institute of Health Research Senior Investigators. Williams is supported by the National Institute of Health Research University College London Hospitals Biomedical Research Centre. Caulfield is supported by the National Institute of Health Research Cardiovascular Biomedical Research Unit at St Bartholomew's Hospital, London. McCann is supported by National Institute of Health Research Research Fellowship.

## Disclosures

Williams has received honoraria from Novartis, Boehringer Ingelheim, Daiichi Sankyo and Servier. McInnes has received honoraria from Novartis. Webb has received funding for membership of Independent Data Monitoring Committees for Abbvie in relation to clinical trials in diabetic nephropathy. Webb is President of the British Pharmacological Society and a Board Member of Medicines and Healthcare products Regulatory Agency. MacDonald is Chief investigator on 2 large investigator initiated, industry‐funded but University sponsored cardiovascular outcome studies (funded by Pfizer and Menarini/IPSEN/Teijin pharmaceuticals). His research unit also does industry funded studies by Novartis and Amgen but none of these studies focus on blood pressure. He has provided consultancy or received honoraria for speaking from Novartis, Takeda, Daiichi Sankyo, Shire and Astellus. Brown has provided external consultancy for GlaxoSmithKline. The remaining authors have no disclosures to report.

## Supporting information


**Table S1.** Inclusion and Exclusion Criteria
**Table S2.** Home and Clinic Systolic and Diastolic BP by Study Week: Least Squares Means (95% Confidence Intervals) Adjusted for Baseline Covariates
**Table S3.** HSBP Results for Primary End Point (Average of Weeks 4–32) and Hierarchical Co–Primary End Point at Week 32 by Morning and Evening Home BP Readings
**Table S4.** Responder and Control Rates
**Table S5.** Initial Monotherapy Patients at Target
**Table S6.** Initial Monotherapy Patients at Target on Their Best Drug
**Table S7.** Initial Monotherapy Patients in Whom Monotherapy was as Effective as or Better Than Combination Treatment
**Table S8.** Adverse Events
**Table S9.** All Significant Adverse Events or Those With Frequency ≥5%
**Table S10.** Reasons for Exclusion From the Per‐Protocol Cohorts
**Figure S1.** Detailed schematic of the PATHWAY‐1 study showing the drug dosing schema used.
**Figure S2.** Time to withdrawal for adverse effects or adverse effects and add‐on therapy, Wilcoxon test (gives greater weight early events): *P*=0.104 for withdrawals only. Log‐rank test (gives greater weight to later events): *P*=0.064 for withdrawals plus add‐on.
**Appendix S1.** PATHWAY study group.
