# Supplementary material for: Combination Therapy Is Superior to Sequential Monotherapy for the Initial Treatment of Hypertension: A Double‐Blind Randomized Controlled Trial
Source: J Am Heart Assoc. 2017 Nov 18;6(11):e006986. doi: 10.1161/JAHA.117.006986 (PMC5721778; doi:10.1161/JAHA.117.006986)
Supplement: Supplementary file 1 — Table S1. Inclusion and Exclusion Criteria Table S2. Home and Clinic Systolic and Diastolic BP by Study Week: Least Squares Means (95% Confidence Intervals) Adjusted for Baseline Covariates Table S3. HSBP Results for Primary End Point (Average of Weeks 4–32) and Hierarchical Co–Primary End Point at Week 32 by Morning and Evening Home BP Readings Table S4. Responder and Control Rates Table S5. Initial Monotherapy Patients at Target Table S6. Initial Monotherapy Patients at Target on Their Best Drug Table S7. Initial Monotherapy Patients in Whom Monotherapy was as Effective as or Better Than Combination Treatment Table S8. Adverse Events Table S9. All Significant Adverse Events or Those With Frequency ≥5% Table S10. Reasons for Exclusion From the Per‐Protocol Cohorts Figure S1. Detailed schematic of the PATHWAY‐1 study showing the drug dosing schema used. Figure S2. Time to withdrawal for adverse effects or adverse effects and add‐on therapy, Wilcoxon test (gives greater weight early events): P=0.104 for withdrawals only. Log‐rank test (gives greater weight to later events): P=0.064 for withdrawals plus add‐on. Appendix S1. PATHWAY study group. [file JAH3-6-e006986-s001.pdf]

Table S1

Inclusion and Exclusion Criteria

Inclusions: (patients must meet all inclusion criteria to be eligible)

1. Aged 18 – 79 years.

2. BP ≥150 mmHg (systolic) OR ≥ 95 mmHg (diastolic) after placebo run-in. Patients may be included if the investigator anticipates BP criteria for inclusion will be met at randomisation (i.e. if BP is likely to meet criteria after withdrawal of previous monotherapy during placebo run-in phase).

3. Either never-treated hypertension or received a maximum of one antihypertensive drug class in the previous year.

4. Male subjects or female subjects taking adequate contraception such as the oral contraceptive pill, an intra uterine device or who are surgically sterilised or postmenopausal females.

Exclusions:

Patients will be excluded for **ANY ONE** of the following reasons

1. Clinic SBP > 200 mmHg or DBP > 120 mmHg, with PI discretion to override if home BP measurements are lower.
2. Secondary or accelerated phase hypertension.
3. eGFR < 45 mls/min.
4. Contra-indication or previous intolerance to any trial therapy.
5. Failure to record required home BP readings during placebo run-in.
6. Significant co-morbidity (investigator opinion but to include alcoholism, terminal illness, documented non-attendance at clinics etc).
7. Diabetes type 1.
8. Plasma K+ outside normal range on two successive measurements during screening.
9. Requirement for treatment with ≥2 drugs (which can be a CCB and/or {ACEi OR ARB OR direct renin inhibitor OR β-blocker}) in order to reduce blood pressure to ≤180/120 mmHg.
10. Requirement for diuretic therapy (other than for hypertension).
11. Requirement for ACE inhibitor (or ARB) therapy (other than for hypertension).
12. Absolute contra-indications to any of the study drugs (listed on their data-sheet).

13. Current therapy for cancer.
14. Anticipation of change in medical status during course of trial (e.g. planned surgical intervention requiring >2 weeks convalescence , actual or planned pregnancy).
15. Inability to give informed consent.
16. Participation in a clinical study involving an investigational drug or device within 4 weeks of screening.
17. Any concomitant condition that, in the opinion of the investigator, may adversely affect the safety and/or efficacy of the study drug or severely limit the subject's lifespan or ability to complete the study (eg, alcohol or drug abuse, disabling or terminal illness, mental disorders).
18. Treatment with any of the following prohibited medications:
a. Oral corticosteroids within 3 months of screening. Treatment with systemic corticosteroids is also prohibited during study participation.
b. Chronic stable or unstable use of non-steroidal anti-inflammatory drugs (NSAIDs) other than acetylsalicylic acid is prohibited. Chronic use is defined as >3 consecutive or nonconsecutive days of treatment per week. In addition, the intermittent use of NSAIDs is strongly discouraged throughout the duration of this study. If intermittent treatment is required, NSAIDs must not be used for more than a total of 2 days. For all subjects requiring analgesic or anti-pyretic agents, the use of paracetamol is recommended during study participation.
c. The use of short-acting oral nitrates (eg, sublingual nitroglycerin) is permitted; however, subjects should not take short-acting oral nitrates within 4 hours of screening or any subsequent study visit.
d. The use of long-acting oral nitrates (eg, Isordil) is permitted; however, the dose must be stable for at least 2 weeks prior to screening and randomisation.
e. The use of sympathomimetic decongestants is permitted; however, not within 1 day prior to any clinic visit/BP assessment.
f. The use of theophylline is permitted; however, the dose must be stable for at least 4 weeks prior to screening and throughout study participation.
g. The use of phosphodiesterase (PDE) type V inhibitors is permitted; however, subjects must refrain from taking these medications within 1 day of screening or any subsequent study visit.
h. The use of alpha-blockers is not permitted – with the exception of afluzosin and tamsulosin for prostatic symptoms.

Table S2

		Blood pressure		Change from baseline		
Home systolic	Week	Combination therapy	Monotherapy	Combination therapy	Monotherapy	Difference
Phase 1	4	136.3 (135.2 to 137.4)	143.0 (141.9 to 144.1)	-15.7 (-16.8 to -14.6)	-9.0 (-10.1 to -7.9)	-6.70 (-8.10 to -5.31)
	8	132.7 (131.to 133.8)	139.8 (138.7 to 140.9)	-19.4 (-20.5, to 18.2)	-12.2 (-13.3 to -11.1)	-7.14 (-8.54 to -5.73)
	12	133.1 (132.0 to 134.3)	141.4 (140.2 to 142.5)	-18.9 (-20.0 to -17.7)	-10.7 (-11.8 to -9.5)	-8.22 (-9.67 to -6.76)
	16	130.2 (129.0 to 131.4)	138.7 (137.5 to 139.9)	-21.9 (-23.1 to -20.7)	-13.3 (-14.5 to -12.1)	-8.53 (-10.1 to -7.00)
Phase 2	24	132.5 (131.4 to 133.7)	132.0 (130.8 to 133.1)	-19.5 (-20.7 to -18.4)	-20.1 (-21.2 to -18.9)	0.52 (-0.93 to 1.98)
	32	130.0 (128.8 to 131.2)	128.4 (127.2 to 129.6)	-22.0 (-23.2 to -20.8)	-23.6 (-24.8 to -22.4)	1.61 (0.07 to 3.15)
Phase 3	38	128.5 (127.4 to 129.6)	128.2 (127.1 to 129.3)	-23.5 (-24.6 to 22.4)	-23.9 (-25.0 to -22.8)	0.35 (-1.03 to 1.74)
	44	128.1 (127.0 to 129.2)	127.0 (125.8 to 128.1)	-23.9 (-25.1 to -22.8)	-25.1 (-26.2 to -24.0)	1.14 (-0.26 to 2.55)
	52	128.4 (127.2 to 129.6)	127.6 (126.4 to 128.8)	-23.6 (-24.8 to -22.4)	-24.5 (-25.6 to -23.3)	0.85 (-0.66 to 2.36)

Home diastolic						
Phase 1	4	85.0 (84.3 to 85.7)	89.4 (88.7 to 90.1)	-8.3 (-9.0 to -7.6)	-3.9 (-4.6 to -3.2)	-4.39 (-5.27 to -3.51)
	8	83.1 (82.4 to 83.8)	87.5 (86.8 to 88.2)	-10.2 (-11.0 to -9.5)	-5.8 (-6.6 to -5.1)	-4.41 (-5.34 to -3.48)
	12	83.0 (82.2 to 83.7)	88.5 (87.8 to 89.3)	-10.4 (-11.1 to -9.6)	-4.8 (-5.5 to -4.0)	-5.59 (-6.56 to -4.62)
	16	81.2 (80.5 to 82.0)	86.8 (86.0 to 87.6)	-12.1 (-12.9 to -11.3)	-6.5 (-7.3 to -5.7)	-5.58 (-6.58 to -4.58)
Phase 2	24	82.7 (82.0 to 83.5)	82.6 (81.9 to 83.4)	-10.6 (-11.4 to -9.8)	-10.7 (-11.5 to -10.0)	0.11 (-0.85 to 1.08)
	32	81.5 (80.7 to 82.2)	80.6 (79.8 to 81.4)	-11.9 (-12.6 to -11.1)	-12.7 (-13.5 to -12.0)	0.88 (-0.12 to 1.87)
Phase 3	38	80.2 (79.5 to 81.0)	80.2 (79.5 to 81.0)	-13.1 (-13.9 to -12.4)	-13.1 (-13.9 to -12.4)	0.00 (-0.96 to 0.96)
	44	80.0 (79.2 to 80.8)	79.6 (78.8 to 80.3)	-13.3 (-14.1 to -12.6)	-13.8 (-14.5 to -13.0)	0.42 (-0.56 to 1.40)
	52	80.0 (79.2 to 80.8)	79.5 (78.7 to 80.2)	-13.4 (-14.2 to -12.6)	-13.9 (-14.7 to -13.1)	0.52 (-0.49 to 1.52)
Clinic systolic						
Phase 1	4	138.7 (137.3 to 140.1)	146.4 (145.0 to 147.9)	-19.1 (-20.5 to -17.7)	-11.4 (-12.8 to -10.0)	-7.71 (-9.49 to -5.93)

	8	134.3 (132.8 to 135.8)	142.9 (141.4 to 144.4)	-23.5 (-25.0 to 22.0)	-14.9 (-16.4 to -13.4)	-8.59 (-10.5 to -6.67)
	12	133.0 (131.5 to 134.6)	145.3 (143.8 to 146.9)	-24.8 (-26.4 to 23.3)	-12.5 (-14.1 to -11.0)	-12.3 (-14.3 to -10.3)
	16	130.2 (128.6 to 131.8)	141.6 (140.0 to 143.2)	-27.6 (-29.2 to -26.0)	-16.3 (-17.8 to -14.7)	-11.4 (-13.4 to -9.31)
Phase 2	24	133.2 (131.6 to 134.8)	133.0 (131.4 to 134.5)	-24.6 (-26.2 to -23.0)	-24.9 (-26.5 to -23.3)	0.27 (-1.78 to 2.32)
	32	129.5 (127.9 to 131.0)	130.2 (128.6 to 131.7)	-28.4 (-29.9 to -26.8)	-27.7 (-29.2 to -26.1)	-0.70 (-2.74 to 1.34)
Phase 3	38	128.8 (127.3 to 130.3)	128.5 (127.0 to 130.0)	-29.1 (-30.6 to -27.5)	-29.3 (-30.8 to -27.8)	0.28 (-1.66 to 2.21)
	44	128.1 (126.7 to 129.6)	127.6 (126.1 to 129.0)	-29.7 (-31.2 to -28.2)	-30.2 (-31.7 to -28.8)	0.55 (-1.32 to 2.42)
	52	128.3 (126.8 to 129.8)	128.4 (126.9 to 129.9)	-29.5 (-31.0 to -28.0)	-29.4 (-30.9 to -27.9)	-0.11 (-2.0 to 1.82)
Clinic diastolic						
Phase 1	4	88.0 (87.0 to 88.9)	93.0 (92.1 to 94.0)	-10.7 (-11.6 to -9.7)	-5.6 (-6.6 to -4.6)	-5.06 (-6.30 to -3.83)
	8	85.3 (84.3 to 86.3)	91.2 (90.2 to 92.2)	-13.3 (-14.3 to -12.3)	-7.4 (-8.4 to -6.4)	-5.90 (-7.15 to -4.64)
	12	84.7 (83.8 to 85.7)	91.9 (91.0 to 92.8)	-13.9 (-14.9 to -12.9)	-6.7 (-7.7 to -5.8)	-7.16 (-8.37 to -5.95)

	16	83.0 (82.0 to 84.0)	90.3 (89.3 to 91.3)	-15.7 (-16.7 to -14.7)	-8.4 (-9.4 to -7.4)	-7.32 (-8.61 to -6.02)
Phase 2	24	85.0 (84.0 to 86.0)	84.8 (83.8 to 85.7)	-13.6 (-14.6 to -12.6)	-13.9 (-14.9 to -12.9)	0.24 (-1.03 to 1.51)
	32	82.8 (81.8 to 83.8)	82.9 (81.9 to 83.9)	-15.8 (-16.8 to -14.8)	-15.7 (-16.7 to -14.7)	-0.12 (-1.42 to 1.18)
Phase 3	38	82.4 (81.4 to 83.4)	82.1 (81.1 to 83.1)	-16.3 (-17.3 to -15.3)	-16.6 (-17.6 to -15.6)	0.29 (-1.00 to 1.58)
	44	81.7 (80.7 to 82.7)	80.9 (79.9, 81.9)	-16.9 (-17.9,-15.9)	-17.8 (-18.8,-16.8)	0.85 (-0.45, 2.15)
	52	81.9 (80.8, 82.9)	81.4 (80.4 to 82.5)	-16.7 (-17.8 to -15.7)	-17.2 (-18.2 to -16.2)	0.46 (-0.90 to 1.81)

Table S3

Home systolic	Blood pressure		Change from baseline			p-value
Mean AM and PM	Combination therapy	Monotherapy	Combination therapy	Monotherapy	Difference	
Average over phases 1 and 2	132·4 (131·4 to 133·4)	137·3 (136·3 to 138·2)	-19·7 (-20·7.to -18·7)	-14·8 (-15·8 to -13·8)	-4·88 (-6·04 to -3·73)	<0·001(1)
End of phase 2	129·8 (128·4 to 131·3)	128·6 (127·2 to 130·0)	-22·2 (-23·6 to -20·8)	-23·4 (-24·8 to -22·0)	1·22 (-0·38 to -2·82)	0·134(2)
Average over phase 1	133.0 (132.0 to 134.0)	141.0 (140.0 to 142.0)	-19.0 (-20.0 to -18.0)	-11.1 (-12.0 to -10.1)	-7.97 (-9.14 to -6.81)	
Average over study (phases 1-3)	131·1 (130·2 to 132·0)	134·0 (133·1 to 134·9)	-20·9 (-21·9 to -20·0)	-18·0 (-18·9 to -17·1)	-2·90 (-3·99 to -1·82)	
AM						
Average over phases 1 and 2	133.1 (132.0 to 134.1)	137.7 (136.7 to 138.8)	-17.9 (-19.0 to -16.8)	-13.2 (-14.3 to -12.2)	-4.65 (-5.89 to -3.41)	<0.001
End of phase 2	130.2 (128.6 to 131.8)	129.5 (127.9 to 131.1)	-20.8 (-22.3 to -19.2)	-21.5 (-23.0 to -19.9)	0.70 (-1.08 to 2.47)	0.440
Average over phase 1	133.7 (132.6 to 134.8)	140.9 (139.9 to 142.0)	-17.2 (-18.3 to -16.2)	-10.0 (-11.1 to -9.0)	-7.20 (-8.45 to -5.96)	
Average over study (phases 1-3)	131.8 (130.8 to 132.8)	134.8 (133.8 to 135.8)	-19.2 (-20.2 to -18.2)	-16.2 (-17.2 to -15.2)	-3.00 (-4.19 to -1.82)	
PM						
Average over phases 1 and 2	131.9 (130.8 to 133.0)	137.4 (136.4 to 138.5)	-21.3 (-22.4 to -20.2)	-15.7 (-16.8 to -14.6)	-5.57 (-6.84 to -4.30)	<0.001
End of phase 2	129.5 (127.9 to 131.0)	127.8 (126.3 to 129.4)	-23.6 (-25.2 to -22.1)	-25.3 (-26.8 to -23.8)	1.64 (-0.12 to 3.39)	0.067
Average over phase 1	132.4 (131.3 to 133.6)	141.4 (140.3 to 142.5)	-20.7 (-21.8 to -19.6)	-11.7 (-12.8 to -10.6)	-8.96 (-10.3 to -7.66)	
Average over study (phases 1-3)	130.6 (129.6 to 131.6)	133.9 (132.9 to 134.9)	-22.5 (-23.6 to -21.5)	-19.3 (-20.3 to -18.3)	-3.28 (-4.46 to -2.10)	

Table S4

	Combination therapy			Monotherapy				
	Patients	Met target		Patients	Met target			
**Controlled**(*)	**n**	**r**	**r/n (%)**	**n**	**r**	**r/n (%)**	**Odds ratio (95%CI)**	**p-value**
Phase 1	298	223	74·8	287	114	39·7	4·66 (3·23 to 6·70)	<·001
Phase 2	265	202	76·2	263	206	78·3	0·88 (0·58 to 1·33)	0·5
Phase 3	241	204	84·6	237	193	81·4	1·22 (0·75 to 2·01)	0·4
(*) Home SBP<135 and home DBP<85 or clinic SBP<140 and clinic DBP<90 at final visit of Phase 1								
Responders(†‡)								
Phase 1	297	289	97·3	287	237	82·6	8·05 (3·69 to 17·55)	<·001
Phase 2	264	252	95·5	263	259	98·5	0·33 (0·10 to 1·05)	0·06
Phase 3	240	237	98·8	237	232	97·9	1·70 (0·39 to 7·33)	0·5

(†) Controlled, or fall in home SBP >10, or fall in clinic SBP>10 at any visit in Phase 1

(‡) One patient with no baseline blood pressures could not be evaluated

Odds ratios from logistic regression models adjusted for baseline covariates

Table S5

Monotherapy at weeks 8 and 16		N	<=135mmHg	Change from baseline		Home SBP	
				Mean	95%CI	Mean	95%CI
	week						
HCTZ	8	144	35.3%	-11.6	-13.2, -9.9	140.3	138.1,142.4
	16	143	31.3%	-11.2	-13.0, -9.3	140.5	138.7,142.4
Losartan	8	143	36.4%	-12.0	-13.5, -10.5	139.8	137.9,141.6
	16	144	45.2%	-14.2	-16.0, -12.4	137.4	135.3,139.6

Initial monotherapy patients at target (%), home SBP (mmHg), and change from baseline, at weeks 8 and 16 during Phase 1. The monotherapies crossed over at week 8. (post hoc analysis)

Table S6

Best monotherapy	N	<=135 mmHg	Change from baseline (mmHg)		Home SBP (mmHg)	
			Mean	95%CI	Mean	95%CI
HCTZ	115	46.2% (36.8,55.8)	-16.8	-18.9,-14.8	136.0	133.8,138.2
Losartan	172	52.4% (44.8,60.6)	-16.3	-17.6,-14.9	134.6	133.1,136.1
All	287	50.0% (44.0,56.0)	-16.5	-17.6,-15.4	135.1	133.9,136.4

Initial monotherapy patients at target on their best drug, at either week 4 or week 8 (post-hoc analysis)

Table S7

	Better at week 32 than best at week 4 or 8?						
	No			Yes			p-value
		Plasma renin (mU/L)			Plasma renin (mU/L)		No vs Yes
Initial therapy	N	Mean	SE	N	Mean	SE	
Combination	104	11.41	1.09	169	11.18	1.07	
HCTZ	18	6.25	1.30	114	12.84	1.09	0.0016
Losartan	17	21.67	1.15	115	11.05	1.08	0.0039

Patients in whom initial monotherapy was as effective as, or better than, subsequent combination treatment. These patients lay in the outer tertiles of plasma renin. (post-hoc analysis)

Table S8

	Combination therapy (N=304)		Monotherapy (N=301)		p value
	n	%	n	%	
Dizziness	73	24.0	62	20.6	0.3
Headache	51	16.8	59	19.6	0.4
Nasopharyngitis	48	15.8	39	13.0	0.4
Fatigue	45	14.8	34	11.3	0.2
Cough	23	7.6	24	8.0	0.9
Lethargy	20	6.6	23	7.6	0.6
Pain in extremity	21	6.9	14	4.7	0.3
Back pain	14	4.6	21	7.0	0.2
Nausea	20	6.6	14	4.7	0.4
Diarrhoea	20	6.6	13	4.3	0.3
Arthralgia	17	5.6	19	6.3	0.7
Paraesthesia	17	5.6	12	4.0	0.4
Dyspepsia	16	5.3	3	1.0	0.004
Dizziness postural	16	5.3	12	4.0	0.6
Rash	12	3.9	16	5.3	0.4
Musculoskeletal pain	3	1.0	14	4.7	0.007

Distinct patients reporting adverse events with each preferred term. Terms listed are those that occurred in at least 5% of patients on any treatment, or were significantly different between treatments (exact p-value for Chi-Square test <0.05)

Table S9

		Combination therapy	Monotherapy	p value
Serious adverse events	Any time	10 (3·3%)	10 (3·3%)	1·0
	Phase 1	5 (1·6%)	5 (1·7%)	1.0
	Phase 2	3 (1·1%)	5 (1·9%)	0·5
	Phase 3	2 (0·8%)	0 (0·0%)	0·5
Any adverseevent	Any time	280 (92·1%)	267 (88·7%)	0·2
	Phase 1	232 (76·3%)	214 (71·1%)	0·2
	Phase 2	159 (58·7%)	171 (64·0%)	0·2
	Phase 3	140 (59·1%)	125 (64·0%)	0·4
AEs suggesting hypotension	Any time	101 (33·2)	93 (30·9)	0·5
	Phase 1	76 (25·0)	41 (13·6)	<0·001
	Phase 2	31 (11·4)	48 (18·0)	0·04
	Phase 3	23 (9·7)	33 (14·5)	0·1
Withdrawals for adverse events	Any time	40(13·4%)	26 (9·1%)	0·1
	Phase 1	20 (6·7%)	10 (3·5%)	0·09
	Phase 2	14 (5·2%)	12 (4·5%)	0·8
	Phase 3	6 (2·5%)	4 (1·7%)	0·8

p-values for Fisher's exact tests

Table S10

		Combination therapy	HCTZ first	Losartan first
Excluded from per protocol cohort		79	42	33
Major protocol deviations		7	4	6
Recording of primary outcome incomplete		16	7	7
Withdrawn or lost to follow up		56	31	20
Reasons for withdrawal				
Subject unwilling to continue	Phase 1	6	5	0
	Phase 2	3	5	0
	Phase 3	0	1	1
	Total	9	11	1
Lost to follow up	Phase 1	4	1	1
	Phase 2	0	3	1
	Phase 3	0	1	1
	Total	4	5	3
Adverse Event	Phase 1	11	2	4
	Phase 2	11	6	6
	Phase 3	2	0	0
	Total	24	8	10
Serious Adverse Event	Phase 1	5	1	2
	Phase 2	2	0	0
	Phase 3	0	0	0
	Total	7	1	2
Investigator terminated participation	Phase 1	1	0	0
	Phase 2	4	3	1
	Phase 3	1	0	0
	Total	6	3	1
Other Reason	Phase 1	3	2	1
	Phase 2	3	1	1
	Phase 3	0	0	1
	Total	6	3	3
Total	Phase 1	30	11	8
	Phase 2	23	18	9
	Phase 3	3	2	3
	Total	56	31	20

Appendix S1

Collaborators

1. Anne Schumann, Jo Helmy, Carmela Maniero, Timothy J Burton, Ursula Quinn, Lorraine Hobbs, Jo Palmer (Addenbrooke's Cambridge, UK)
2. David Collier, Nirmala Markandu, Manish Saxena, Anne Zak, Enamuna Enobakhare (William Harvey Institute, QMUL, London, UK)
3. Judith Mackay, Simon A McG Thom, Candida Coughlan, (International Centre for Circulatory Health UCL London, UK)
4. Alison R McGinnis, Evelyn Findlay (Ninewells Hospital & Medical School, Dundee, UK)
5. Adrian G Stanley, Gerry P McCann, Christobelle White, Peter Lacy, Caroline J. Gardiner-Hill, Sheraz Nazir. (Glenfield Hospital, Leicester, UK)
6. Sandosh Padmanabhan, Gordon T McInnes, Scott Muir, Linsay McCallum (Glasgow Cardiovascular Research centre, Glasgow, UK)
7. Vanessa Melville, Iain M MacIntyre, Roger Brown, Simon R (University of Edinburgh Western General Hospital, Edinburgh, UK)
8. Handrean Soran, See Kwok, Karthirani Balakrishnan. (Old Saint Mary's Hospital, Manchester, UK)
9. Richard Hobbs, Rachel Iles. (Primary Care Clinical Sciences University Birmingham, Birmingham, UK)
10. Khin Swe Myint (Clinical Research and trials Unit, University of East Anglia, Norwich, UK)
11. John Cannon, Sue Hood (Ixworth GP Practice, Ixworth, UK)
12. Krzysztof Rutkowski, Andrew Webb (Cardiovascular Medicine & Diabetes, King's College London, London, UK)
13. Una Martin (University Hospital Birmingham, Birmingham, UK)
14. Sharon Kean, Robbie Wilson, Richard Papworth (Robertson Centre for Biostatistics and Glasgow CTU, Glasgow, UK)

Figure S1

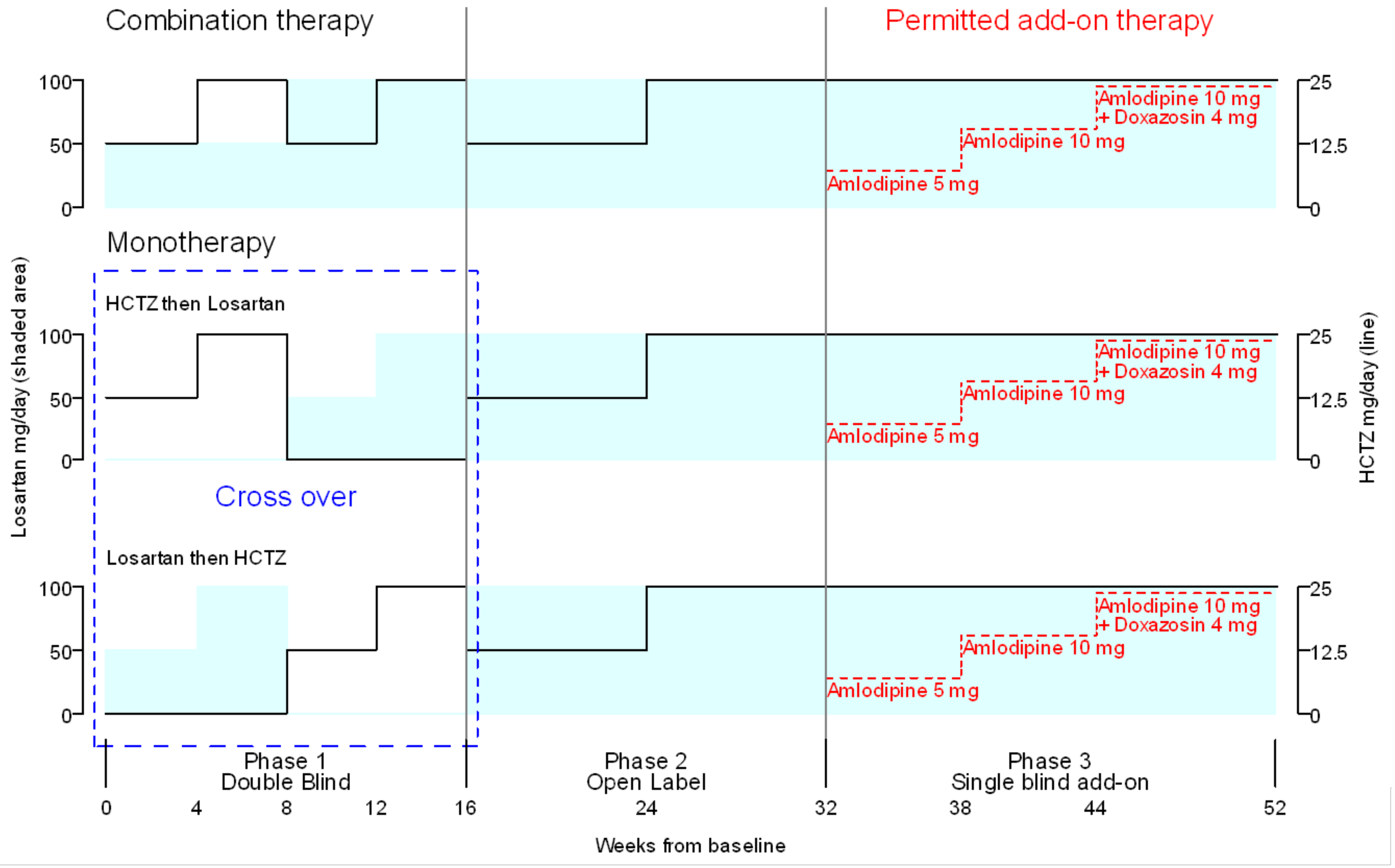

Detailed schematic of the PATHWAY 1 study showing the drug dosing schema used.

Figure S2

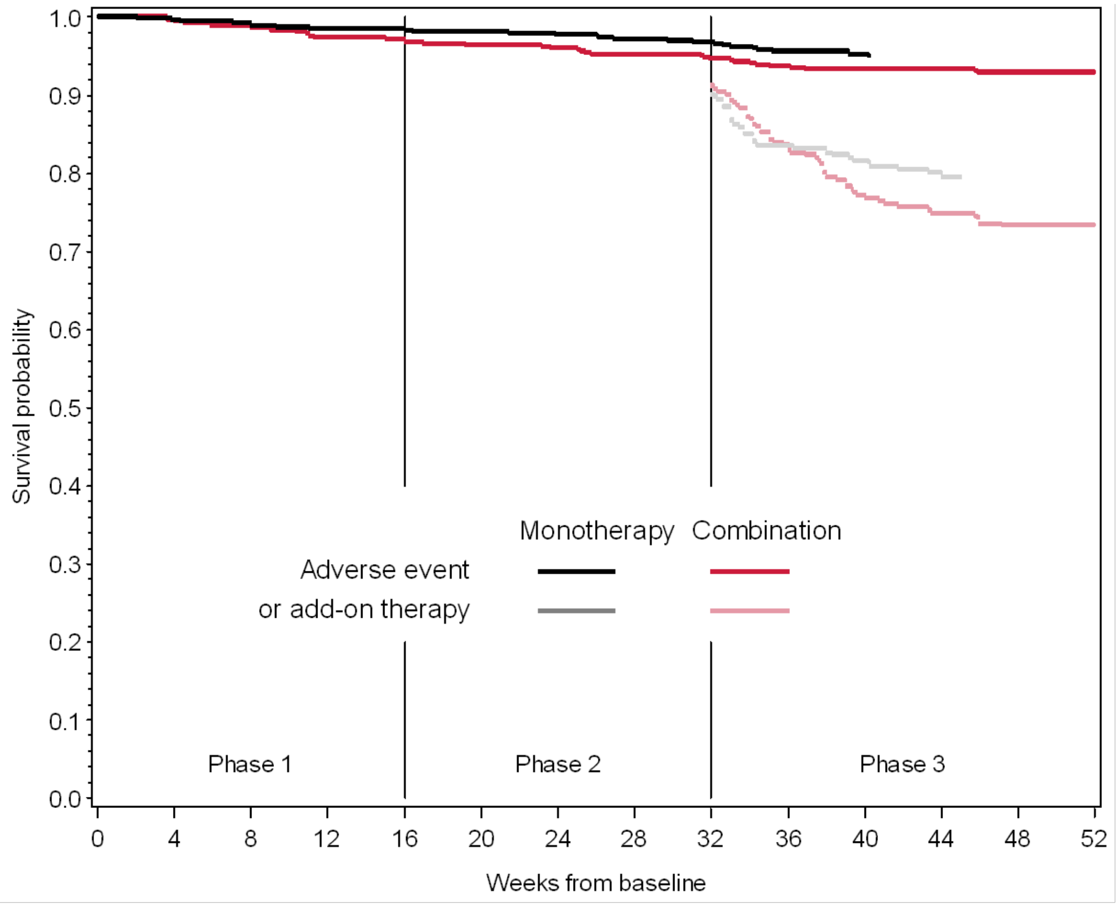

Kaplan-Meier plots for time to adverse event, or time to the earlier of adverse event or add on therapy

Wilcoxon test (gives greater weight early events): p=0.104 for withdrawals only. Log-rank test (gives greater weight to later events): p=0.064 for withdrawals + add-on
